# Inferior Trapezius Myocutaneous Flap as Salvage Procedure for Large Posterior Scalp Defect

**Published:** 2015-07-28

**Authors:** Adam M. Feintisch, Angie M. Paik, Ramazi Datiashvili

**Affiliations:** Division of Plastic Surgery, Rutgers UniversityUniveristy – New Jersey Medical School, Newark, NJ

**Keywords:** trapezius, scalp defect, myocutaneous, pedicled flap, reconstruction

## DESCRIPTION

A 53-year-old male presented status post resection of recurrent meningiomas with an open posterior scalp wound, 18 × 14 cm, with exposed calvarium and titanium mesh used for cranioplasty.

## QUESTIONS

1. **What are the reconstructive options for a posterior scalp defect?**
2. **What are the possible variations of trapezius myocutaneous flaps for head and neck reconstruction?**
3. **What skin paddle dimensions can be obtained with an inferior trapezius myocutaneous flap?**
4. **What is the arc of rotation of an inferior trapezius myocutaneous flap?**

## DISCUSSION

When evaluating any scalp defect, important considerations include the size and depth of the defect, the presence of periosteum, bone defect, prior irradiation, and any preexisting scars. The reconstructive options for a posterior scalp defect can usually be determined on the basis of the size of the wound. Small defects (<2 cm^2^) can be closed primarily, medium defects (2–25 cm^2^) may be reconstructed using rotation-advancement flaps, and large defects (>25 cm^2^) can be reconstructed with larger rotation flaps.^1^ Pedicled or free tissue transfers may be required to provide coverage for large defects as well. Some of the notable options include a latissimus dorsi free flap, a pedicled trapezius myocutaneous flap, an anterolateral thigh flap, or an omental flap.^2^^-^6

The trapezius muscle and the overlying skin are supplied by the occipital artery superiorly, the transverse cervical artery (TCA) in the mid-portion of the muscle, and the dorsal scapular artery inferiorly. The trapezius muscle is classically described having a type II vascular pattern, with the TCA as the dominant pedicle. Despite this, standard cadaveric studies have shown great vascular variability where both the TCA and the dorsal scapular artery have been shown to be the dominant pedicle and may explain the flap's unpredictable results.^7^^,^^8^ While several variations in flap design have been described in the literature, the major flap options include the superior, lateral, and lower trapezius myocutaneous flaps.^9^ The superior flap can be used for coverage of the posterolateral neck and is especially valuable after radical neck dissection and to cover irradiated wounds. The lateral flap is used for external defects of the lateral and anterior neck as well as mucosal defects of the pharynx and the oral cavity. The lower trapezius myocutaneous flap, as utilized in our case, has the most versatility in clinical application and has the most use for reconstruction of the occiput.^8^

The lower trapezius myocutaneous flap pedicle is designed with the TCA as its pedicle. For this reason, the flap has typically been centered with the long axis lying between the vertebral column and the medial border of the scapula. The maximum flap length has been reported as 38 cm, with the cranial border extended as far superiorly as the spine of the scapula to preserve shoulder function. The caudal extent of the flap can extend past the lower border of the muscle by 10 to 13 cm.^10^^,^^11^ While there have been no formal studies on the acceptable width of the lower trapezius myocutaneous flap, clinical studies have reported a wide range of 7 to 22 cm.^11^^,^^12^

The superficial branch of the TCA lies superficial to the levator scapulae and rhomboid muscles. This permits a wide arc of rotation without tethering when the pivot point is centered at the posterior base of the neck. Coverage may be achieved as far cephalad as the vertex of the skull. The parieto-occipital scalp, lateral face, and neck can also be covered without tension.^13^

**Figure 1:**
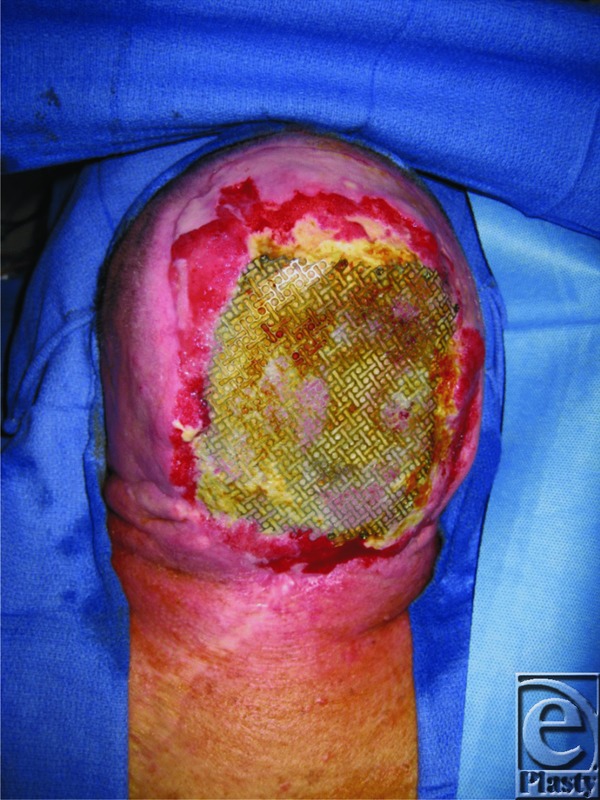
Open wound of the posterior scalp with exposed titanium mesh.

**Figure 2:**
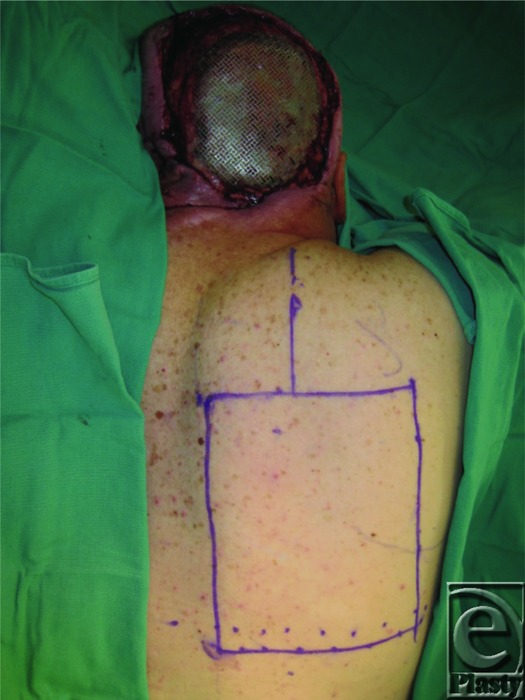
An 18 × 14-cm skin paddle designed over the lower part of the trapezius muscle.

**Figure 3:**
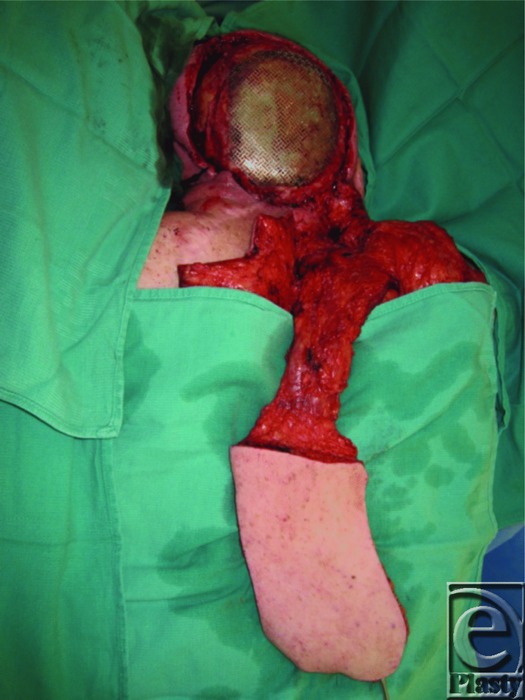
The mobilized trapezius myocutaneous flap.

**Figure 4:**
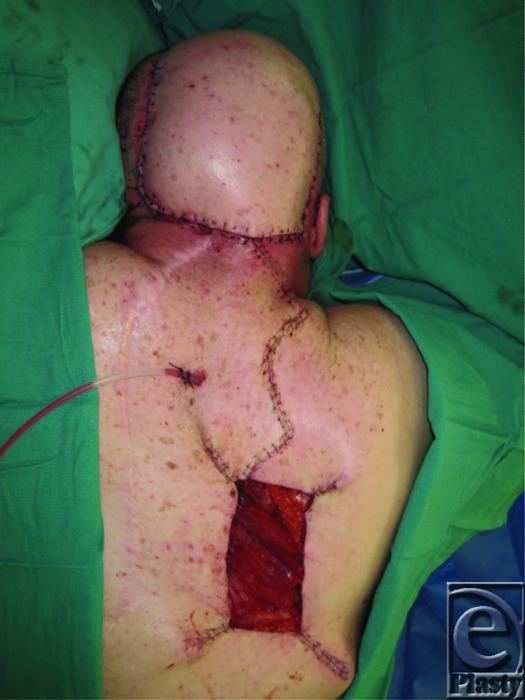
Immediate postoperative view after flap inset.

**Figure 5:**
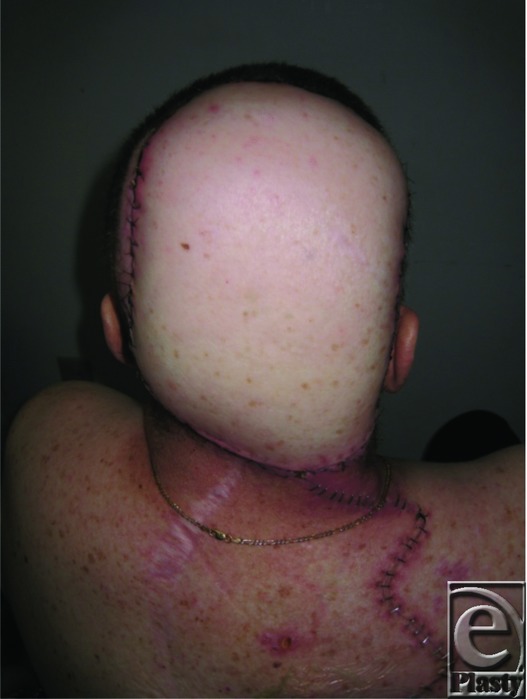
Two weeks after the surgery.

We present the case of a 53-year-old man with a history of multiple resections for recurrent meningiomas. The last ablating surgery left him with an 18 × 14-cm open posterior scalp wound with exposed titanium mesh (Fig 1). The decision was made to use a lower trapezius myocutaneous flap for reconstruction of the defect. An 18 × 14-cm skin paddle was designed with the cranial border at the level of mid-scapula, the caudal border roughly at T12, the medial border running 2 cm lateral to spinous processes, and the lateral border at the neck of the scapula. The flap was mobilized and inset over the defect (Figs 2–4). The donor site wound was partially closed, and the remaining 12 × 10-cm defect was initially managed with negative pressure therapy. Subsequently, it was covered with a split-thickness skin graft. There were no postoperative complications (Fig 5). The flap and the skin graft completely survived. Our case describes the successful use of a lower trapezius myocutaneous flap to cover a large posterior scalp defect. In our experience, this flap is able to sustain a substantial skin paddle, with lateral borders as far as 14 cm from the vertebral column. This flap also has a wide arc of rotation that permits coverage to the posterior scalp as far as the vertex.
